# Pivotal Roles of GM-CSF in Autoimmunity and Inflammation

**DOI:** 10.1155/2015/568543

**Published:** 2015-03-08

**Authors:** Aoi Shiomi, Takashi Usui

**Affiliations:** Department of Rheumatology and Clinical Immunology, Graduate School of Medicine, Kyoto University, Kyoto 606-8507, Japan

## Abstract

Granulocyte macrophage-colony stimulating factor (GM-CSF) is a hematopoietic growth factor, which stimulates the proliferation of granulocytes and macrophages from bone marrow precursor cells. In autoimmune and inflammatory diseases, Th17 cells have been considered as strong inducers of tissue inflammation. However, recent evidence indicates that GM-CSF has prominent proinflammatory functions and that this growth factor (not IL-17) is critical for the pathogenicity of CD4^+^ T cells. Therefore, the mechanism of GM-CSF-producing CD4^+^ T cell differentiation and the role of GM-CSF in the development of autoimmune and inflammatory diseases are gaining increasing attention. This review summarizes the latest knowledge of GM-CSF and its relationship with autoimmune and inflammatory diseases. The potential therapies targeting GM-CSF as well as their possible side effects have also been addressed in this review.

## 1. Introduction

Granulocyte macrophage colony-stimulating factor (GM-CSF) is a hematopoietic growth factor which was originally recognized as a stimulator for the proliferation of granulocytes and macrophages from bone marrow precursor cells [1]. It has also been shown to promote the survival and activation of mature myeloid cells and therefore contributes to the maintenance of innate immune homeostasis [2]. Recent studies suggest that GM-CSF also has proinflammatory functions and plays critical roles in the development of autoimmune and inflammatory diseases, particularly in Th17 driven diseases [3, 4].

## 2. Biology of GM-CSF

### 2.1. Production of GM-CSF

GM-CSF is produced by a variety of cells. Major sources of GM-CSF include activated T and B cells, monocytes/macrophages, endothelial cells, fibroblasts, and other sources such as neutrophils, eosinophils, epithelial cells, mesothelial cells, chondrocytes, Paneth cells, and tumor cells [5–7]. The production of GM-CSF in T cells is stimulated by IL-1*β* and IL-23 in mice [3, 8], IL-1*β* and IL-12 in humans [9], and also prostaglandin E2 [10]. In fibroblasts, endothelial cells, chondrocytes, and smooth muscle cells, it is stimulated by TNF-*α* and IL-1, and in macrophage/monocytes it is stimulated by toll like receptors (TLRs) [5]. In lymphocytes, the transcription factor nuclear factor of activated T cells (NFAT) is reported to be required for the production of GM-CSF [11, 12]. However, the production of GM-CSF can be inhibited by IFN-*γ* [13], IL-4 [14], IL-10 [15], and also pharmacological agents such as cyclosporine A [16, 17] or glucocorticoids [18].

### 2.2. GM-CSF Receptor and Signaling

The GM-CSF receptor is expressed on myeloid cells and on some nonhaemopoietic cells such as endothelial cells but not on T cells [19, 20]. The GM-CSF receptor is a heterodimer of an *α*-subunit which binds GM-CSF with low affinity and a signaling *β*c-subunit which is shared with the IL-3 and IL-5 receptors [21]. The *β*c-subunit constitutively associates with Janus kinase 2 (JAK2) and is tyrosine phosphorylated by it resulting in an assembly of dodecameric signaling complex and initiation of signaling [22]. The effects of GM-CSF are mediated in a dose-dependent manner, by two *β*-chain residues: Ser585 and Tyr577 [23]. At low concentrations of GM-CSF, as in normal healthy tissues, signaling occurs via Ser585 of the *β*-chain, which leads to activation of the PI-3 kinase pathway and results in myeloid cell survival. At high concentrations of GM-CSF, as at the site of inflammation, signaling via Ser585 is extinguished and signaling occurs exclusively via Tyr577 residue, which activates the Jak2/STAT5 pathway, Ras/mitogen-activated protein kinase pathway and PI-3 kinase pathway, resulting in cell survival, proliferation, and activation [23–26].

### 2.3. Action of GM-CSF

GM-CSF stimulates proliferation and activation of macrophages, monocytes, neutrophils, eosinophils, dendritic cells, and microglia [1, 27]. However, since GM-CSF-deficient mice did not have a defect in myeloid cell development [28], a redundant role of GM-CSF in myeloid cell development and differentiation under steady state or homeostatic conditions is predicted. In addition to its function as a hematopoietic growth factor, GM-CSF is now recognized to have a variety of functions on mature hemopoietic cells. GM-CSF enhances proinflammatory cytokine production [29], antigen presentation [30, 31], and phagocytosis [32–35] and promotes leukocyte chemotaxis and adhesion [5, 36–38]. GM-CSF deficient mice have increased susceptibility to pulmonary [28, 39–41] and intestinal infections [42] followed by systemic infection, indicating its importance in maintaining immune homeostasis particularly in the lung and intestines, which are constitutively exposed to pathogens.

#### 2.3.1. Macrophages

GM-CSF stimulates the terminal differentiation of macrophages and the acquisition of normal immune functions via the transcription factor PU.1 [32]. GM-CSF also regulates phagocytosis of microbial pathogens by macrophages through the upregulation of pathogen associated molecular pattern (PAMP) receptors such as C-type lectins including mannose receptors or Dectin-1, scavenger receptors, integrins, or Fc*γ* receptors via PU.1 [24, 32–34, 43]. Complement-dependent phagocytosis is also enhanced by GM-CSF to control microbial pathogens [44]. GM-CSF also upregulates the expression of TLR2, TLR4, or CD14 and boosts the production of proinflammatory cytokines such as TNF, IL-6, IL-12p70, IL-23, or IL-1*β* [24, 32, 45, 46], leading to polarization of macrophages to the M1- (classic-) like phenotype, thus, promoting Th1–Th17 responses [29, 47, 48] and contributing to tissue destruction [49]. On the other hand, M-CSF polarizes macrophages to the M2- (alternative-) like phenotype, which produces anti-inflammatory cytokines such as IL-10 and CC-chemokine ligand 2 (CCL2) and promotes tissue repair and remodeling [49]. GM-CSF also regulates many functions in macrophages including cell adhesion [32], pulmonary surfactant lipid and protein catabolism [32], and several important antimicrobial activities such as the production of reactive oxygen species (ROS) or expression of antimicrobial enzymes [40].

#### 2.3.2. Dendritic Cells (DCs)

GM-CSF positively regulates the development of migratory CD103^+^CD11b^+^ DCs [50] but negatively regulates the development of resident CD8^+^ DCs [51]. GM-CSF also strongly induces the development of inflammatory monocyte-derived DCs (moDCs)* in vitro* [52]. However, it has not been well established whether GM-CSF also regulates the development of moDCs* in vivo*. It was reported that the number of moDCs was increased in GM-CSF transgenic mice [53]. Furthermore, NF-*κ*B1-dependent GM-CSF production in CD4 T cells was reported to be required for the generation of moDCs in inflammatory arthritis and antigen-induced peritonitis mouse models. The number of moDCs was markedly reduced in draining lymph nodes from GM-CSF−/− mice with inflammatory arthritis or in the spleen of mice reconstituted with NF-*κ*B1−/− CD4 T cells in acute peritonitis, demonstrating that GM-CSF contributes to the differentiation of these cells during inflammation* in vivo* [54]. On the other hand, GM-CSF was shown to be dispensable for the differentiation of moDCs, at least during acute infections, since the number of moDCs was not decreased in GM-CSF−/− mice or GM-CSF receptor deficient mice during acute infections [55, 56]. These data indicate that although GM-CSF strongly regulates the production of moDCs* in vitro* and* in vivo*, there may be another GM-CSF-independent pathway for the development of moDCs [56]. Besides the regulation of DC development, GM-CSF also upregulates cross-presentation, bacterial uptake [53], or production of proinflammatory cytokines such as IL-6 or IL-23 in resident DCs [57].

#### 2.3.3. Neutrophils

In mature neutrophils, GM-CSF upregulates the expression of the integrin CD11b, which increases cellular adhesion and tissue entry [36]. GM-CSF also upregulates the antimicrobial functions of neutrophils, such as phagocytosis or ROS production [58]. However, the expression of PU.1 in neutrophils of autoimmune pulmonary alveolar proteinosis patients was normal, indicating that GM-CSF is not involved in neutrophil differentiation [58].

#### 2.3.4. B Cells

Among B cells, the innate-like B1 B cells reside predominantly in serosal cavities such as the pleural or peritoneal cavity. In response to microbial infection, B1a B cells (a subset of B1 B cells) recognize bacteria via direct TLR-dependent pathogen recognition and differentiate into innate response activator (IRA) B cells, which produce GM-CSF and also express the GM-CSF receptor [59, 60]. GM-CSF acts on its receptor in an autocrine manner and induces IgM production from B cells [59, 61]. Mixed chimeric mice with B cell-restricted GM-CSF deficiency showed high bacterial titer and morbidity after infection but did not show alveolar proteinosis [60], indicating that B cell-derived GM-CSF is necessary for protective IgM responses but dispensable for surfactant clearance by alveolar macrophages. These data indicate that the cellular source and location of GM-CSF is important.

## 3. T Cell and GM-CSF

**Figure 1:**
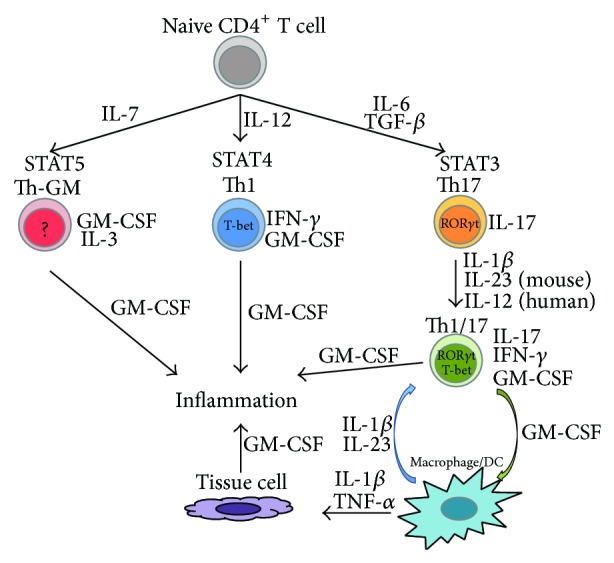
The differentiation of GM-CSF-producing CD4^+^ T cells and cytokine networks. Activated macrophages and dendritic cells (DCs) produce proinflammatory cytokines such as IL-23, IL-1*β*, and IL-6, which can promote the differentiation of Th17 and Th1/17 cells, leading to more GM-CSF production from Th1/17 cells. Increased levels of GM-CSF upregulate further production of proinflammatory cytokines from macrophages/DCs, creating a positive feedback loop. Activated macrophages/DCs also produce proinflammatory cytokines, such as IL-1*β* and TNF*α*, which stimulate the production of GM-CSF from resident tissue cells, including endothelial cells, epithelial cells, fibroblast, or chondrocytes.

Although GM-CSF is widely expressed in both stromal and hematopoietic cells, recent murine studies suggest that GM-CSF from CD4^+^ T cells is essential in inflammatory mouse models such as experimental autoimmune encephalomyelitis (EAE), arthritis models such as collagen-induced arthritis (CIA) or SKG-arthritis, interstitial lung disease in SKG mice (SKG-ILD), peritonitis, or myocarditis [3, 4, 54, 57, 62–64]. Although GM-CSF is known as one of the Th17 cytokines, Th1 cells and Th2 cells also express GM-CSF [64–67]. Moreover, recent studies represent the existence of GM-CSF-producing Th cells distinct from Th1, Th2, or Th17 cells [62, 64] (Figure 1).

### 3.1. Th17 and Th1/17 Cells

Th17 cells have been shown to be strong inducers of tissue inflammation and autoimmune diseases. However, a number of studies determined that IL-17 inhibition does not prevent but rather only ameliorates the development of EAE [3, 4, 68, 69], CIA [70], SKG-arthritis, or SKG-ILD [63] and that neutralizing IL-17 is a rather unsatisfactory method for blocking Th17 mediated diseases [71, 72]. Recently, it was reported that “classical” Th17 cells, which mainly produce IL-17, are not pathogenic and that Th17 cells have high plasticity. As such, they instead differentiate into highly pathogenic cells, called Th1/17 cells [73]. Th1/17 cells are characterized by their ability to coproduce IL-17, IFN-*γ*, and GM-CSF and are identified by the coexpression of T-bet, ROR*γ*t, and the chemokine receptors CXCR3 and CCR6 [9, 74–78]. Human Th1/17 cells also express CD161, a hallmark of Th17 progeny cells in humans that is induced by ROR*γ*t [76, 79, 80]. Recent studies report that GM-CSF is critical for the pathogenicity of Th17 cells [3, 4] and the presence of Th1/17 cells was observed at the inflammatory site of inflammatory bowel disease (IBD), multiple sclerosis (MS), and juvenile idiopathic arthritis (JIA) [76, 78, 81, 82].

In mice, IL-23 and IL-1*β* induce the production of GM-CSF in T cells whereas IL-12 suppresses its expression [3, 4, 77, 83]. In contrast, in humans IL-1*β* renders Th17 cells sensitive to IL-12 and both IL-1*β* and IL-12 promote the differentiation of Th1/17 cells [9, 75, 81, 84, 85] (Figure 1). As described in Section 2.3, GM-CSF induces the differentiation of M1-like macrophages and upregulates the production of proinflammatory cytokines such as IL-6, IL-12, IL-23, or IL-1*β* from antigen presenting cells (APCs) [57]. This results in further differentiation of Th17 and Th1/17 cells, thus creating a positive feedback loop [3, 63].

Studies show that GM-CSF expression in CD4^+^ T cells is not regulated by T-bet [3, 4] and ROR-responsive elements are identified in the promoter of the gene encoding GM-CSF [4]. Moreover, ectopic ROR*γ*t expression in CD4^+^ T cells results in GM-CSF production, indicating that GM-CSF production in Th1/17 cells is induced by ROR*γ*t [4]. Conversely, ROR*γ*t-deficient CD4^+^ T cells can produce GM-CSF, indicating the existence of additional pathways to induce GM-CSF production in CD4^+^ T cells [3].

### 3.2. Th-GM Cells

Recently, it was reported that IL-2- or IL-7-activated STAT5 promotes the generation of GM-CSF-producing CD4^+^ T cells with low or undetectable expression of T-bet, GATA-3, ROR*γ*t transcripts. These cells represent a new distinct subset of Th cells, namely, Th-GM [62, 64]. In humans, these Th-GM cells are identified as CCR10^+^CCR4^+^CXCR3^−^CCR6^−^ Th cells [64]. It was reported that IL-7-activated STAT5 directly bound to promoter regions of the gene encoding GM-CSF [62]. The contribution of IL-7 has been implicated in autoimmune diseases such as multiple sclerosis or rheumatoid arthritis [86, 87], which also suggests the contribution of Th-GM cells in these diseases. High expression of IL-3, which is also involved in several autoimmune diseases [88–90], was also reported in Th-GM cells [62]. These cells were reported to be able to induce a more severe experimental autoimmune encephalomyelitis (EAE) than Th17 or Th1 cells [62, 64]. It is possible that Th-GM cells provide GM-CSF to induce the expression of IL-23 from APCs and cooperate with Th1/17 or Th1 cells to exacerbate the development of inflammation.

### 3.3. Th1 Cells

Th1 cells are another source of GM-CSF [4]. It was reported that Th1 cells also need GM-CSF to mediate inflammation in the central nervous system (CNS) [4]. However, the amount of GM-CSF produced by Th1 cells is found to be consistently lower than that produced by Th17 cells, particularly during* in vitro* culture [65].

### 3.4. Th2 Cells

Th2 cells also produce GM-CSF [66, 67]. Although a positive correlation was found between GATA-3^+^ cells and GM-CSF^+^ cells in the nasal mucosa of patients with allergic rhinitis [91], there is no study to our knowledge that directly analyzes the role of GATA-3 in GM-CSF production. Further investigation is therefore needed to elucidate the precise mechanism of GM-CSF production from CD4^+^ T cells and their contribution to the development of autoimmune and inflammatory diseases.

## 4. GM-CSF in Autoimmune and Inflammatory Diseases

### 4.1. GM-CSF in Central Nervous System

Multiple sclerosis (MS) is a chronic inflammatory disease of the central nervous system and is pathologically characterized by demyelination and subsequent axonal degeneration. Past studies have shown that CD4^+^ T cells play a critical role in the development of MS and experimental autoimmune encephalomyelitis (EAE), a widely used mouse model of MS. It has been widely believed that Th17 cells are the main encephalitogenic population in autoimmune inflammation [92]; however, IL-17 has been found to be dispensable for the development of EAE [4, 93]. On the other hand, GM-CSF deficiency or neutralization of GM-CSF has been reported to prevent the onset of EAE [94, 95]. The administration of recombinant GM-CSF worsened the disease in EAE [94] and elevated concentrations of GM-CSF have been reported in the cerebrospinal fluid but not in the serum of patients with relapsing-remitting or secondary progressive MS [96, 97].

Recent findings show that GM-CSF is secreted by CNS-infiltrating helper T cells and is essential for encephalitogenicity in EAE [3, 4, 64]. GM-CSF induces the proliferation and activation of microglial cells, which is required for the onset of EAE [95, 98]. Activated microglial cells produce highly neurotoxic substances such as ROS, nitrogen species, glutamate, and TNF-*α* [99–102]. Furthermore, GM-CSF-producing CD4^+^ T cells induce the differentiation of neurotoxic M1-like phenotype of microglia [103] and upregulate the production of proinflammatory mediators such as IL-1*β*, IL-6, and TNF*α*, which contribute to myelin sheath damage, via upregulation of TLR and CD14 expression [45].

Studies also show that GM-CSF is required for recruitment of peripheral myeloid cells that contribute to blood-brain barrier (BBB) and blood-spinal cord barrier (BSCB) disruption and demyelization into the CNS [104, 105]. As we described in Section 3.1, GM-CSF induces the polarization of the M1-like macrophage phenotype and exacerbates the positive feedback loop of Th17 and Th1/17 differentiation. Indeed, an increased M1/M2 profile ratio of monocyte/macrophages in the blood as well as in the CNS favors relapsing EAE and a reduced M1/M2 ratio promotes an attenuated manifestation of the disease [106]. The ability of CNS-invading myeloid cells to respond to CD4^+^ T cell-derived GM-CSF was shown to be vital for the development of EAE [4].

Taken together, CNS-infiltrating CD4^+^ T cells initially activate microglia and induce production of proinflammatory cytokines, which contribute to myelin sheath damage [95]. This initial neuroinflammation results in BBB destruction and leukocyte infiltration into the CNS parenchyma, followed by restimulation of T cells by resident and infiltrating APCs [107], leading to further APC activation.

These reports indicate that GM-CSF plays a central role in MS and indicate that the inhibition of GM-CSF will be a useful therapeutic strategy for MS. MOR103, a fully human monoclonal antibody that binds human GM-CSF, is currently being tested in a phase Ib trial for MS [108] (Table 1). The result of this and future trials are highly anticipated.

### 4.2. GM-CSF in Arthritis

Rheumatoid arthritis (RA) is a systemic chronic autoimmune disease characterized by persistent and erosive inflammatory polyarthritis. Recent studies indicate that GM-CSF plays a central role in the pathogenesis of RA as in MS, by activating or promoting differentiation and survival of macrophages and neutrophils [109, 110]. The concentrations of GM-CSF were elevated in the synovial fluid and plasma of RA patients [111, 112]. A case report showed that the administration of recombinant GM-CSF exacerbated the disease activity of RA [113]. The frequency of GM-CSF-producing Th cells was significantly increased in synovial fluid cells compared to peripheral blood mononuclear cells (PBMCs) in patients with juvenile idiopathic arthritis (JIA) and correlated with erythrocyte sedimentation rate (ESR) levels [81, 114]. Synovial GM-CSF-producing T cells were predominantly CD161 positive and coexpressed IFN-*γ* but not IL-17 [81], indicating that these cells are Th1/17 cells. Alternatively, human synovial fibroblasts and chondrocytes were also reported to produce GM-CSF in response to IL-1 and TNF stimulation [115, 116].

The contribution of GM-CSF in the development of arthritis was also reported in several mouse models of arthritis. In the collagen-induced arthritis (CIA) model, GM-CSF deficient mice failed to develop arthritis [117], and the administration of anti-GM-CSF neutralizing antibodies ameliorated existing disease, prevented disease progression, and reduced the concentrations of TNF and IL-1 in the joints of treated mice [118]. On the other hand, GM-CSF administration exacerbated arthritis in CIA [119].

In SKG mice, another model of autoimmune arthritis, GM-CSF, upregulated proinflammatory cytokine production such as IL-1*β* or IL-6 from macrophages in a dose dependent manner [63, 120]. This in turn induced further differentiation and expansion of IL-17-producing and GM-CSF-producing CD4^+^ T cells [63]. The progression of arthritis in SKG mice was inhibited by the neutralization of GM-CSF and slightly by the neutralization of IL-17A [63], indicating that GM-CSF plays a more critical role than IL-17A in SKG arthritis.

Mavrilimumab, a fully human anti-GM-CSF receptor *α* antibody, is currently being developed and a phase II study in RA patients reported significant efficacy with no serious adverse events such as pulmonary alveolar proteinosis [121]. In this study of patients with active RA despite methotrexate treatment, 55.7% of all participants treated with mavrilimumab met the primary end point of achieving ≥1.2 decrease from baseline in the disease activity score (DAS28-CRP) at week 12. At the highest dose of mavrilimumab (100 mg), 66.7% of subjects met the primary end point versus only 34.7% of the subjects in the placebo group [121].

MOR103, which is also being tested for MS, has shown preliminary evidence of efficacy in a phase Ib/IIa trial for patients with active RA [122]. Subjects receiving higher doses of MOR103 (1.0 and 1.5 mg/kg) showed significant improvement in DAS28 scores and joint counts and significantly higher European league against rheumatism response rates than subjects receiving placebo [122]. Both mavrilimumab and MOR103 showed rapid treatment responses and provided evidence of clinical efficacy that support further clinical investigation.  Table 2 shows the results of clinical trials with mavrilimumab and MOR103. A list of ongoing/completed clinical trials targeting GM-CSF or its receptor is presented in Table 1, with more information available at ClinicalTrials.gov.

### 4.3. GM-CSF in Lung Disease

#### 4.3.1. Pulmonary Alveolar Proteinosis

Pulmonary alveolar proteinosis (PAP) is a rare syndrome characterized by the accumulation of surfactant in pulmonary alveoli resulting in varying degrees of respiratory insufficiency and myeloid cell dysfunction leading to increased risk of infection [123, 124]. Several clinical forms of PAP have been identified including autoimmune PAP caused by GM-CSF autoantibodies, hereditary PAP caused by GM-CSF receptor mutations, and secondary PAP associated with various underlying clinical disorders which is presumed to cause this syndrome by reducing alveolar macrophage numbers or function [125]. It is also reported that GM-CSF deficient mice develop abnormal lung histology that is virtually indistinguishable from human PAP [126].

Pulmonary surfactant is tightly regulated by balanced production, secretion, reuptake, and catabolism within alveoli. GM-CSF regulates surfactant catabolism in alveolar macrophages via PU.1 but does not regulate surfactant endocytosis or uptake and catabolism of surfactant by alveolar epithelial cells type II (AEC-II) [32, 127]. GM-CSF is also required to stimulate numerous immune functions and terminal differentiation of alveolar macrophages [32] and induction of IgM production from B cells [60, 61]. Therefore, GM-CSF deficient mice have high susceptibility to pulmonary infections [28, 39–41] accompanied by systemic infections. Based on the pathogenesis of PAP, several new therapeutic approaches for treating autoimmune PAP targeting GM-CSF are in clinical trials, including plasmapheresis [128], GM-CSF administration [129, 130], and rituximab [131, 132].

#### 4.3.2. Interstitial Lung Disease

The mechanism of pulmonary fibrosis in interstitial lung disease (ILD) has been studied using Idiopathic pulmonary fibrosis (IPF) or a bleomycin-induced mouse model of pulmonary fibrosis. Pulmonary fibrosis in IPF is considered to be inflammation-independent and mainly initiated by TGF-*β* produced by damaged epithelial cells. In fact, anti-inflammatory therapies have little benefit in IPF [133–135]. However, neutrophils, which produce ROS, MMPs, neutrophil elastase, or myeloperoxidase and cause lung parenchymal and stromal cell injury [136–138] or Th2 cytokines such as IL-4 or IL-13, which induce fibroblast differentiation or extracellular matrix synthesis [139, 140], have been reported to contribute to lung fibrosis to some extent. Additionally, GM-CSF was reported to take part in the progress of pulmonary fibrosis. It was reported that TNF*α*-induced endothelin-1 (ET-1) upregulates GM-CSF production from airway smooth muscle cells (ASMCs) [141] and that GM-CSF was increased in the bronchoalveolar lavage fluid (BALF) of patients with pulmonary fibrosis [142, 143]. GM-CSF stimulates macrophages to release profibrotic cytokines and also induces fibrosis by direct stimulation of airway smooth muscle cells [144, 145]. In fact, overexpression of GM-CSF in the lungs led to severe neutrophil, eosinophil, and macrophage infiltration and fibrotic reactions [145–147].

In contrast with idiopathic pulmonary fibrosis (IPF), connective tissue disease-associated ILD (CTD-ILD) is often characterized by a clearer response to immunosuppression, indicating that autoimmune/inflammatory mechanisms play a more significant and central role in the pathogenesis of CTD-ILD [148, 149]. SKG mice, a model of autoimmune arthritis, were reported to develop chronic-progressive interstitial lung disease (ILD) that histologically resembles CTD-ILD [63, 150, 151]. Recently, it was reported that ILD in this mouse was characterized by massive infiltration of Th17 cells, GM-CSF-producing CD4^+^ T cells, and CD11b^+^Gr1^+^ neutrophils with fibrosis [63]. Naive SKG T cells were skewed to differentiate into GM-CSF-producing cells. Furthermore, GM-CSF secreted by T cells enhanced IL-6 and IL-1*β* production by macrophages, which in turn enhanced differentiation of IL-17A and/or GM-CSF-producing T cells and infiltration of neutrophils into the lungs [63]. Neutralization of GM-CSF completely blocked the development of this ILD, whereas neutralization of IL-17A did not, showing that GM-CSF not IL-17A is critical for the development of ILD in SKG mice [63]. Importantly, neutralization of GM-CSF ameliorated ILD in SKG mice even after the onset of ILD [63], suggesting that GM-CSF inhibition is a useful therapeutic strategy for CTD-ILD. Mavrilimumab, a fully human anti-GM-CSF receptor *α* antibody and MOR103, a fully human monoclonal anti-GM-CSF antibody, are undergoing clinical trials in RA patients [121, 122]. Further studies and future trials targeting GM-CSF are awaited with interest.

### 4.4. GM-CSF in Intestine

Recent developments suggest that the development of Crohn's disease (CD) is caused by a mucosal innate immunodeficiency with a variety of genetic defects [152] and a dysfunction of granulocytes, macrophages, and intestinal epithelial cells [153, 154]. In the intestine, GM-CSF contributes to gut barrier function and resistance to bacterial translocation by promoting the recruitment and activation of monocytes/macrophages, neutrophils, and DCs. This is accompanied by differentiation of Th1 and Th17 cells. GM-CSF also promotes tissue repair via increased intestinal epithelial cell proliferation and increased macrophages as effectors of wound healing [155–157]. However, in CD patients, the inherent defects in the mucosal barrier increase the translocation of pathogens to the bowel tissue [152]. Moreover, increased levels of GM-CSF autoantibodies have been reported in patients with ileal/ileocolonic CD, compared with those with colon involvement only, ulcerative colitis (UC) patients, or healthy controls. The high levels of GM-CSF antibodies directly correlated with disease activity and inversely correlated with neutrophil phagocytic activity [158]. Increased GM-CSF autoantibodies affect mucosal integrity, bacterial killing and neutrophil migration, proliferation, and survival [156]. GM-CSF deficient mice also developed a more severe intestinal and systemic infection after enteric infection [42] and were more susceptible to acute dextran sodium sulfate- (DSS-) induced colitis [159]. Both severity of infection and colitis were largely prevented by GM-CSF administration [160, 161]. On the other hand, GM-CSF overexpression in the stomach leads to autoimmune gastritis [162] and experimental peritonitis or intraperitoneal LPS exposure in GM-CSF deficient mice resulted in blunted proinflammatory responses and mortality [163]. The expression of GM-CSF at the mRNA level increases from the stomach distally down through the colon, indicating that gastrointestinal expression of GM-CSF expression parallels bacterial localization [164]. These data indicate that the threshold of GM-CSF levels between inflammation and immune homeostasis can vary with tissue location or organs.

These findings indicate that administration of GM-CSF could be beneficial for the treatment for CD patients. Initial reports indicated that the administration of GM-CSF in CD patients with moderate to severe disease activity had a high rate of clinical response and remission with minimal adverse effects [165, 166]. Moreover, a randomized phase II clinical trial demonstrated that GM-CSF was significantly more effective than placebo in obtaining a corticosteroid-free clinical remission [167]. Conversely, a recent large randomized trial found that it was no more effective than placebo for induction of clinical remission or improvement in active CD [168]. CD patients are known to have substantial heterogeneity in pathogenic mechanisms and so GM-CSF as a therapy may only be appropriate for a subgroup of patients. Additionally, it is also important to verify the efficacy of GM-CSF not only for the induction of remission but also for the maintenance of remission. Therefore, further studies are needed to elucidate the efficacy of GM-CSF as a treatment as well as the appropriate patient character or phase of disease to apply GM-CSF administration as a therapy.

### 4.5. GM-CSF in Allergic Disease

GM-CSF also was reported to take part in Th2 response in allergic airway inflammation via activation of DCs [169–171]. In mouse models of asthma, allergen-exposed epithelial cells release GM-CSF which activates DCs and also prolongs eosinophil survival [170, 172]. Consequently, GM-CSF neutralization reduced allergic hyperresponsiveness in mice models [169, 170, 172]. KB003, a “humaneered” anti-GM-CSF antibody, is tested in a phase II trial for severe asthma (Table 1). The result of this trial is awaited.

## 5. Conclusion

GM-CSF plays pivotal roles not only in maintaining immune homeostasis but also in exacerbating inflammatory reactions. Recent findings indicate that GM-CSF inhibition will be an attractive therapeutic strategy for many autoimmune and inflammatory diseases. Studies also indicate that it is necessary to monitor possible side effects such as PAP or CD, although GM-CSF inhibition has no demonstrated serious adverse reactions so far, which is indicative of its wide therapeutic index [63, 173, 174]. Further studies are necessary to identify the molecular mechanisms that regulate GM-CSF production and the role of GM-CSF in the development of inflammatory diseases to devise preventive or curative strategies for autoimmune and inflammatory diseases.

## Figures and Tables

**Table 1:** Clinical trials targeting GM-CSF.

Target	Drug	Type	Indication	Phase	Status
GM-CSFR	Mavrilimumab (CAM-3001)	mAb	RA	II	Completed
GM-CSF	MOR103	mAb	RA MS	Ib/IIa Ib	Completed Completed, not published yet
GM-CSF	Namilumab (MT203)	mAb	RA Plaque psoriasis	I II	Completed, not published yet On-going
GM-CSF	KB003	Humaneered mAb	RA Asthma	II II	Terminated, not published yet Completed, not published yet
GM-CSF	MORAb-022	mAb	RA	I	On-going

GM-CSFR: GM-CSF receptor; mAb: monoclonal antibody; RA: rheumatoid arthritis; MS: multiple sclerosis.

**Table 2:** Effects of GM-CSF inhibitors in RA.

Drug	Regimen	Patients	Efficacy (versus placebo)
Mavrilimumab (CAM-3001) Phase II [121]	10, 30, 50, or 100 mg, SC every other week	*N* = 233 On stable MTX	DAS28 change: −1.2 at week 12 41.0%, 61.0%, 53.8%, and 66.7% versus 34.7% (*P* = 0.543, 0.011, 0.071, and 0.001)
			ACR at week 12 100 mg dose versus Placebo ACR20: 69.2% versus 40.0% (*P* = 0.005) ACR50: 30.8% versus 12.0% (*P* = 0.021) ACR70: 17.9% versus 4.0% (*P* = 0.030)

MOR103 Phase Ib/IIa [122]	0.3, 1.0, or 1.5 mg/kg, IV once a week for 4 weeks, with follow-up to 16 weeks	*N* = 96	Significant differences in DAS28 change between placebo and MOR-103: None, weeks 4 through 10, weeks 4 through 6
			ACR at week 4, 1.0 mg/kg versus placebo ACR20: 68.2% versus 7.4% (*P* < 0.0001) ACR50: 22.7% versus 3.7% (ns) ACR70: 4.5% versus 0.0% (ns)

SC: subcutaneous injection; IV: intravenous injection; MTX: methotrexate; DAS28: 28-joint disease activity score; ACR20: American College of Rheumatology 20% response rate; ACR50: American College of Rheumatology 50% response rate; ACR70: American College of Rheumatology 70% response rate.
